# Efficacy of platelet-rich plasma or plasma rich in growth factors in the treatment of thin endometrium: a systematic review and meta-analysis of randomized controlled trials

**DOI:** 10.3389/fendo.2026.1888268

**Published:** 2026-08-17

**Authors:** Xiaodan Yin, Mengyuan Li, Qian Han, Siqi Guan, Junqin He

**Affiliations:** Department of TCM, Beijing Obstetrics and Gynecology Hospital, Capital Medical University, Beijing Maternal and Child Health Care Hospital, Beijing, China

**Keywords:** assisted reproduction, clinical pregnancy, endometrial thickness, meta-analysis, plasma rich in growth factors, platelet-rich plasma, thin endometrium

## Abstract

**Background:**

Thin endometrium is a recognized cause of implantation failure in assisted reproduction, and conventional treatments are frequently disappointing. Intrauterine infusion of autologous platelet-rich plasma (PRP) or plasma rich in growth factors (PRGF) has emerged as a candidate therapy, but the randomized evidence remains limited and inconsistent.

**Objective:**

To synthesize the randomized controlled evidence on the efficacy of intrauterine PRP or PRGF for women with thin endometrium, with clinical pregnancy as the primary outcome and biochemical pregnancy, endometrial thickness change, and cycle cancellation as secondary outcomes.

**Design:**

Systematic review and random-effects meta-analysis of randomized controlled trials (RCTs).

**Methods:**

PubMed, EMBASE, the Cochrane Library, and Web of Science were searched from inception to 30 April 2026. RCTs comparing intrauterine PRP or PRGF against a non-PRP control in women with thin endometrium were eligible. Risk of bias was appraised with the Cochrane RoB 2 tool and certainty of evidence with GRADE. Dichotomous outcomes were pooled as risk ratios (RR) and endometrial thickness change as a mean difference (MD), using random-effects models with the Hartung-Knapp adjustment. Heterogeneity was quantified with the I-squared statistic, and leave-one-out sensitivity analyses were performed.

**Results:**

Seven RCTs were included. For clinical pregnancy, five trials (343 participants) yielded a pooled RR of 2.32 (95% CI 1.02-5.31; p=0.047; I-squared=23.0%). PRP or PRGF significantly increased endometrial thickness (six trials, 370 participants; MD 0.94 mm, 95% CI 0.34-1.55; p=0.010) but with very high heterogeneity (I-squared=90.5%). Biochemical pregnancy (four trials, RR 2.32, 95% CI 0.71-7.52; p=0.108) and cycle cancellation (three trials, exploratory; RR 0.44, 95% CI 0.00-203.51; p=0.622) did not differ significantly. Leave-one-out analysis showed that the clinical-pregnancy result lost statistical significance when any of several trials was removed. Certainty of evidence was very low for pregnancy and thickness outcomes.

**Conclusion:**

Intrauterine PRP or PRGF reliably increases endometrial thickness and shows a marginally significant, fragile improvement in clinical pregnancy, without demonstrable benefit for biochemical pregnancy or cycle cancellation. Given the small number of trials, high risk of bias, and very low certainty of evidence, these findings should be regarded as preliminary and require confirmation in larger, rigorously designed RCTs.

**Systematic review registration:**

https://www.crd.york.ac.uk/PROSPERO/home, identifier CRD420261383681.

## 1 Introduction

Successful embryo implantation in assisted reproductive technology (ART) requires a developmentally competent embryo and a receptive endometrium of adequate thickness (1). Endometrial thickness, measured by transvaginal ultrasonography, is the most widely used surrogate marker of endometrial receptivity in ART cycles. Although its discriminatory value for predicting pregnancy has been questioned, and live births have been reported across a wide range of thicknesses, an unusually thin endometrium is consistently associated with lower implantation and clinical-pregnancy rates and is a frequent reason for cycle cancellation (1, 2). In routine practice, thin endometrium is most often defined as an endometrial thickness below 7 mm on the day of progesterone initiation or embryo transfer, although stricter thresholds are also used (2, 3). Thin endometrium is relatively uncommon—fewer than 5% of women in large ART cohorts have a lining below 7 mm—but when it is refractory to conventional treatment it is disproportionately difficult to manage and is an important cause of endometrial-factor infertility (2). The causes of a persistently thin endometrium are heterogeneous and include prior intrauterine surgery or curettage, intrauterine adhesions (Asherman syndrome), chronic endometritis, prolonged use of clomiphene citrate, radiotherapy, and, in many women, no identifiable cause. Structurally, a thin lining is often characterized by reduced glandular epithelium, high uterine artery impedance with poor sub-endometrial blood flow, and diminished vascular endothelial growth factor expression, which together compromise the endometrial response to estrogen. Because these features reflect both structural loss and functional impairment, interventions that address only one dimension may be insufficient, which helps explain why no single treatment has proved reliably effective.

A range of interventions has been tried for thin endometrium that is refractory to standard estrogen therapy, including extended or high-dose estrogen administration, vasodilators such as low-dose aspirin, pentoxifylline and vitamin E, granulocyte colony-stimulating factor, and intrauterine instillation of various growth-factor preparations; none has proved consistently effective, and the management of refractory thin endometrium remains an unmet clinical need (3). Against this background, autologous platelet-rich plasma (PRP) has attracted growing interest as a regenerative option. PRP is a concentrate of autologous plasma in which platelets are enriched to roughly three-to-five times the physiological level; upon activation, the platelet alpha-granules release a rich array of growth factors and cytokines—including platelet-derived growth factor (PDGF), vascular endothelial growth factor (VEGF), transforming growth factor-beta (TGF-beta), epidermal growth factor (EGF), and insulin-like growth factor-1 (IGF-1)—that together can promote endometrial epithelial and stromal proliferation, angiogenesis, and tissue regeneration (4–6). Because it is autologous, PRP carries minimal risk of disease transmission or immunogenic and allergic reactions, which is part of its appeal for repeated intrauterine use (6). Plasma rich in growth factors (PRGF) is a closely related autologous preparation with a comparable rationale, and the specific method of PRP preparation and activation—centrifugation force, leukocyte content, and activation agent—can itself influence the concentration and release kinetics of these factors, a source of variability that has complicated cross-study comparison (7). PRP has an established role in other fields of regenerative medicine, and this track record has motivated its translation to reproductive medicine. In the reproductive setting it has been applied not only to thin endometrium but also to recurrent implantation failure and to poor ovarian response, reflecting a broader interest in autologous regenerative approaches to improving reproductive outcomes.

The first report of intrauterine PRP for thin endometrium described successful endometrial expansion and pregnancy in a small series of women undergoing *in vitro* fertilization (IVF) (4). Subsequent case series and observational studies have generally reported favorable effects on endometrial thickness and pregnancy (5, 8–11), and several randomized controlled trials (RCTs) have since been conducted (12–17). However, these trials are small, heterogeneous in design and clinical setting, and inconsistent in their conclusions, and the largest trial in the field—which found no benefit of PRP over placebo—has so far appeared only as a conference abstract (18). Previous meta-analyses have addressed intrauterine PRP in assisted reproduction more broadly (19, 20), but the specific question of its efficacy in thin endometrium, incorporating the most recent RCTs and a formal appraisal of the certainty of evidence, warrants a dedicated and up-to-date synthesis. A focused synthesis restricted to randomized trials in thin endometrium, analyzing clinically distinct outcomes separately and formally grading the certainty of evidence, is therefore needed to inform both clinical counseling and the design of future trials.

We therefore performed a systematic review and meta-analysis of RCTs to evaluate the efficacy of intrauterine PRP or PRGF in women with thin endometrium, taking clinical pregnancy as the primary outcome and biochemical pregnancy, endometrial thickness change, and cycle cancellation as secondary outcomes, and grading the certainty of the resulting evidence.

## 2 Materials and methods

This systematic review was conducted and reported in accordance with the Preferred Reporting Items for Systematic Reviews and Meta-Analyses (PRISMA) 2020 statement (21). The protocol was registered in PROSPERO [PROSPERO registration number: CRD420261383681].

### 2.1 Search strategy and information sources

We systematically searched PubMed, EMBASE, the Cochrane Library, and Web of Science from database inception to 30 April 2026. The search combined controlled vocabulary and free-text terms, with the core concepts being “platelet-rich plasma,” “plasma rich in growth factors,” “PRP,” “PRGF,” “thin endometrium,” “endometrial thickness,” and “embryo transfer,” adapted to the syntax of each database. The reference lists of included articles were hand-searched to identify additional eligible studies. Records were managed and de-duplicated in EndNote.

### 2.2 Eligibility criteria

#### 2.2.1 Inclusion criteria

Studies were eligible if they (1) were RCTs; (2) enrolled infertile women whose endometrium remained thin after standard estrogen therapy (most commonly defined as an endometrial thickness below 7 mm); (3) used intrauterine infusion or injection of autologous PRP or PRGF as the intervention; (4) compared this against a non-PRP control, such as estrogen therapy alone, a sham procedure, or placebo; and (5) reported at least one of the following outcomes: clinical pregnancy, biochemical pregnancy, endometrial thickness change, cycle cancellation, or live birth.

#### 2.2.2 Exclusion criteria

We excluded (1) non-randomized studies, including retrospective studies, prospective cohorts, and single-arm series; (2) trials lacking a non-PRP control arm, such as those comparing a single versus a double PRP infusion; (3) studies available only as conference abstracts for which complete data could not be obtained; and (4) duplicate publications or reports from which data could not be extracted.

### 2.3 Data extraction and handling of endometrial thickness change

Two reviewers independently screened records and extracted data, with disagreements resolved by discussion or adjudication by a third reviewer. Extracted items included the first author, year of publication, country, study design, clinical setting, sample size, intervention and control protocols, event counts, and mean ± standard deviation (SD) for continuous outcomes. For PRP/PRGF protocols, we additionally extracted blood-draw volume, centrifugation method, platelet concentration, leukocyte content, activation method, infused volume, number and timing of administrations, and administration route; items not reported in the source article were recorded as not reported.

A specific methodological point concerns the SD of the change in endometrial thickness. Most included trials reported pre- and post-treatment endometrial thickness with their SDs but did not directly report the SD of the change score. For these studies (Nazari 2019, Eftekhar 2018, Pandey 2023, and Abduljabbar 2022), we imputed the SD of the change using the method recommended in the Cochrane Handbook (22), assuming a within-patient correlation of 0.5 between pre- and post-treatment measurements; Pothisan 2025 and Castells 2025 reported change scores and their SDs directly and were used as reported. A sensitivity analysis varying the correlation between 0.3 and 0.7 did not alter the direction or statistical significance of the pooled result.

### 2.4 Risk of bias and certainty of evidence

Methodological quality was assessed with the revised Cochrane risk-of-bias tool for randomized trials (RoB 2) across five domains: the randomization process (D1), deviations from intended interventions (D2), missing outcome data (D3), measurement of the outcome (D4), and selection of the reported result (D5), together with an overall judgment (23). The certainty of evidence for each main outcome was rated with the GRADE approach as high, moderate, low, or very low (24).

### 2.5 Statistical analysis

Data were pooled and visualized in R using the meta (25) and metafor (26) packages. Dichotomous outcomes (clinical pregnancy, biochemical pregnancy, and cycle cancellation) were summarized as risk ratios (RR), and the continuous outcome (endometrial thickness change) as a mean difference (MD), each with its 95% confidence interval (CI). Because the number of trials was small and their samples modest, a random-effects model with the Hartung-Knapp adjustment to the CI of the pooled estimate was used throughout (27). Between-study heterogeneity was quantified with the I-squared statistic. Robustness was examined by leave-one-out sensitivity analysis. Where enough trials were available, Egger’s and Begg’s tests were used to explore small-study effects (28), but because fewer than 10 trials contributed to every outcome these results are reported only as exploratory. The significance level was set at α=0.05. PRP and PRGF were pooled under a pragmatic class-effect assumption because both are autologous platelet-derived preparations intended to deliver concentrated platelet growth factors to the endometrium; this assumption did not imply that the products were biologically identical, and robustness was examined in a PRP-only sensitivity analysis excluding the PRGF trial. FET, IVF/ICSI, and OS-IUI trials were pooled because all enrolled women with thin endometrium and evaluated the same endometrium-directed intervention and clinical-pregnancy outcome; random-effects models were used and clinical setting was examined in a prespecified subgroup analysis. A non-significant subgroup test was not interpreted as proof of equivalence. Live birth was not meta-analysed because only two RCTs reported it and the data were too sparse for a reliable pooled estimate, so it was summarized narratively.

## 3 Results

### 3.1 Study selection and characteristics of included studies

**Figure 1:**
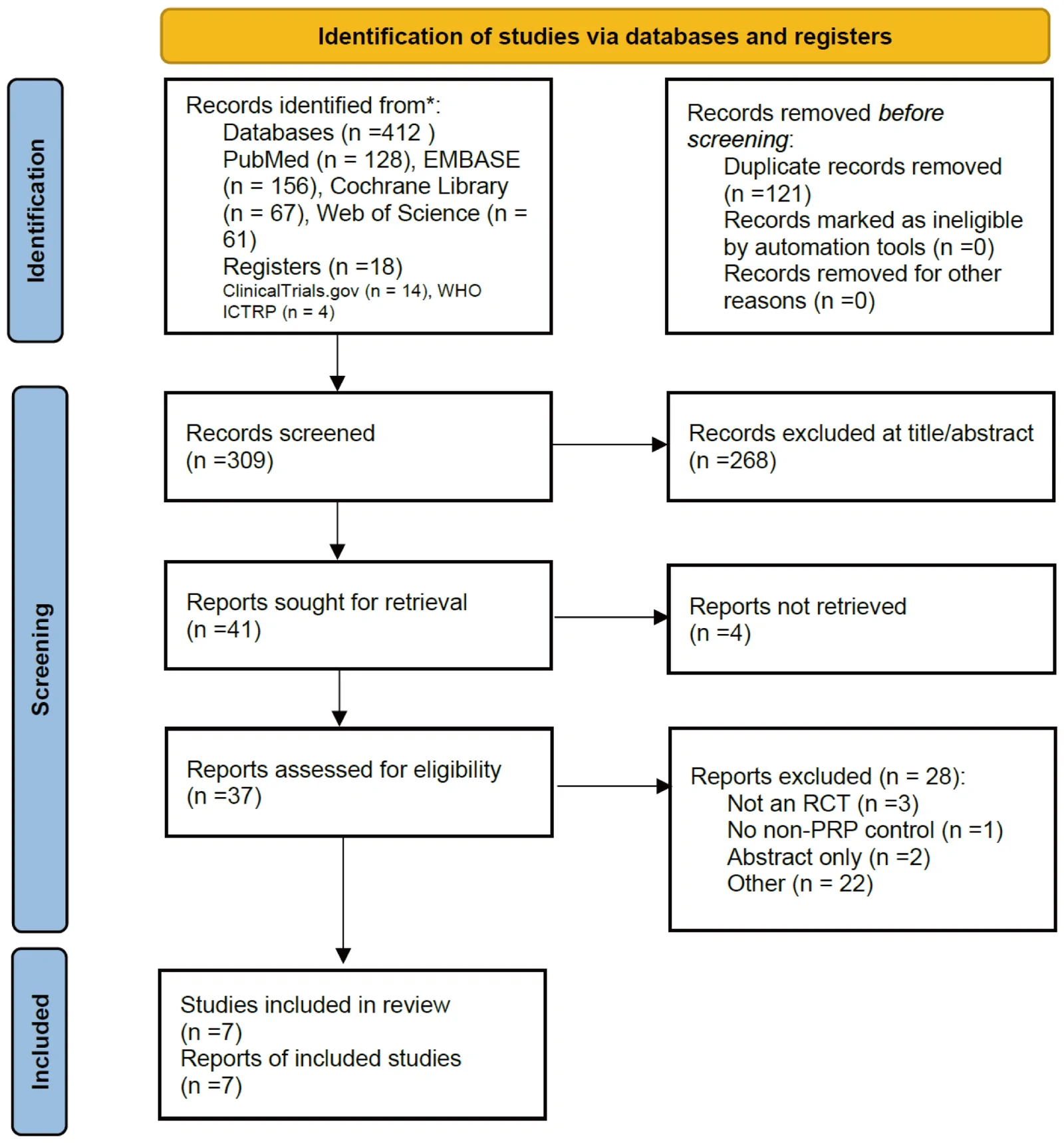
PRISMA 2020 flow diagram of study identification, screening, and inclusion.

The database and hand searches yielded the records summarized in the PRISMA flow diagram (**Figure 1**). After stepwise screening, seven RCTs were included in the quantitative synthesis (12–16, 29, 30).

The included trials were conducted in Iran, Thailand, Saudi Arabia, India, Spain, and Russia, and spanned three clinical settings: frozen-thawed embryo transfer (FET), IVF/intracytoplasmic sperm injection (IVF/ICSI), and ovarian stimulation with intrauterine insemination (OS-IUI). Nazari 2019 was a double-blind, sham-controlled trial and was methodologically the strongest (12). Castells 2025 used PRGF and, because no control participant reached embryo transfer, contributed only to the endometrial-thickness analysis (29). Efendieva 2023 differed methodologically by using hysteroscopically guided intraendometrial PRP injection with a physiotherapy comparator and was therefore reserved for sensitivity analysis (30). **Table 1** summarizes the study characteristics together with the reported PRP/PRGF preparation and administration protocols, which varied substantially among trials.

**Table 1:** Characteristics and PRP/PRGF preparation and administration protocols of the included randomized controlled trials.

Study (Year)	Country	Design	Setting	N (PRP)	N (Ctrl)	TE threshold (mm)	Product	PRP/PRGF preparation	Administration
Nazari (2019) (12)	Iran	Double-blind sham RCT	FET	30	30	7	PRP	Blood draw (mL): Not reported; Centrifugation: Two-step centrifugation (details referenced to pilot protocol); Platelet concentration: Not reported; Leukocytes: Not reported; Activation: Not reported	Volume (mL): 0.5; No. of administrations: 2; Timing: HRT/FET cycle day 11-12; second infusion 48 h later; Route: Intrauterine IUI catheter under ultrasound guidance
Eftekhar (2018) (13)	Iran	Open-label RCT	FET	40	43	7	PRP	Blood draw (mL): 8.5; Centrifugation: 1600 g 10 min + 3500 g 5 min; Platelet concentration: Approximately 4–5 fold baseline; Leukocytes: Buffy coat collected; Activation: Not reported	Volume (mL): 0.5-1.0; No. of administrations: 1-2; Timing: HRT cycle day 13; Route: IUI catheter
Pothisan (2025) (15)	Thailand	Single-blind RCT	FET	10	8	7	PRP	Blood draw (mL): 15; Centrifugation: 1800 rpm 12 min + 3400 rpm 7 min; Platelet concentration: 4–5 fold baseline; Leukocytes: Not reported; Activation: Activated; activator not reported	Volume (mL): 1; No. of administrations: 1; Timing: FET cycle day 8; Route: Intrauterine IUI catheter under transabdominal ultrasound guidance
Abduljabar (2022) (14)	Saudi Arabia	Non-blind RCT	IVF/ICSI	35	35	7	PRP	Blood draw (mL): 10-20; Centrifugation: 1500 rpm 10 min + 3000 rpm 5 min; Platelet concentration: Not reported; Leukocytes: Not reported; Activation: Not reported	Volume (mL): 0.5; No. of administrations: 1; Timing: After oocyte pickup; Route: Intrauterine IUI catheter
Pandey (2023) (16)	India	Open-label RCT	OS-IUI	59	58	7	PRP	Blood draw (mL): 13.5; Centrifugation: 1500 rpm 15 min + 3000 rpm 15 min; Platelet concentration: 4–5 fold baseline; Leukocytes: Leukocyte-poor pure PRP; Activation: Not reported	Volume (mL): 0.5-1.0; No. of administrations: Up to 3 cycles; Timing: Ovulation stimulation cycle; Route: IUI catheter
Castells (2025) (29)	Spain	RCT + observational	FET	13	9	5	PRGF	Blood draw (mL): 18; Centrifugation: 580×g 8 min; Platelet concentration: Not reported; Leukocytes: Buffy coat avoided; Activation: Calcium chloride 10%	Volume (mL): 0.5; No. of administrations: 3; Timing: Repeated administrations during cycle; Route: Embryo transfer catheter
Efendieva (2023)* (30)	Russia	Pilot randomised	IVF	42	30	7	PRP	Blood draw (mL): Not reported; Centrifugation: Not reported in main article; Platelet concentration: 0.6-0.7×10^11 platelets per preparation; Leukocytes: Not reported; Activation: Non-activated PRP	Volume (mL): 35-40; No. of administrations: 1; Timing: Proliferative phase, cycle days 6-9; PRP instead of conservative therapy; Route: Hysteroscopic intraendometrial injection via endoscopic needle (2–3 mm depth)

### 3.2 Risk of bias

**Figure 2:**
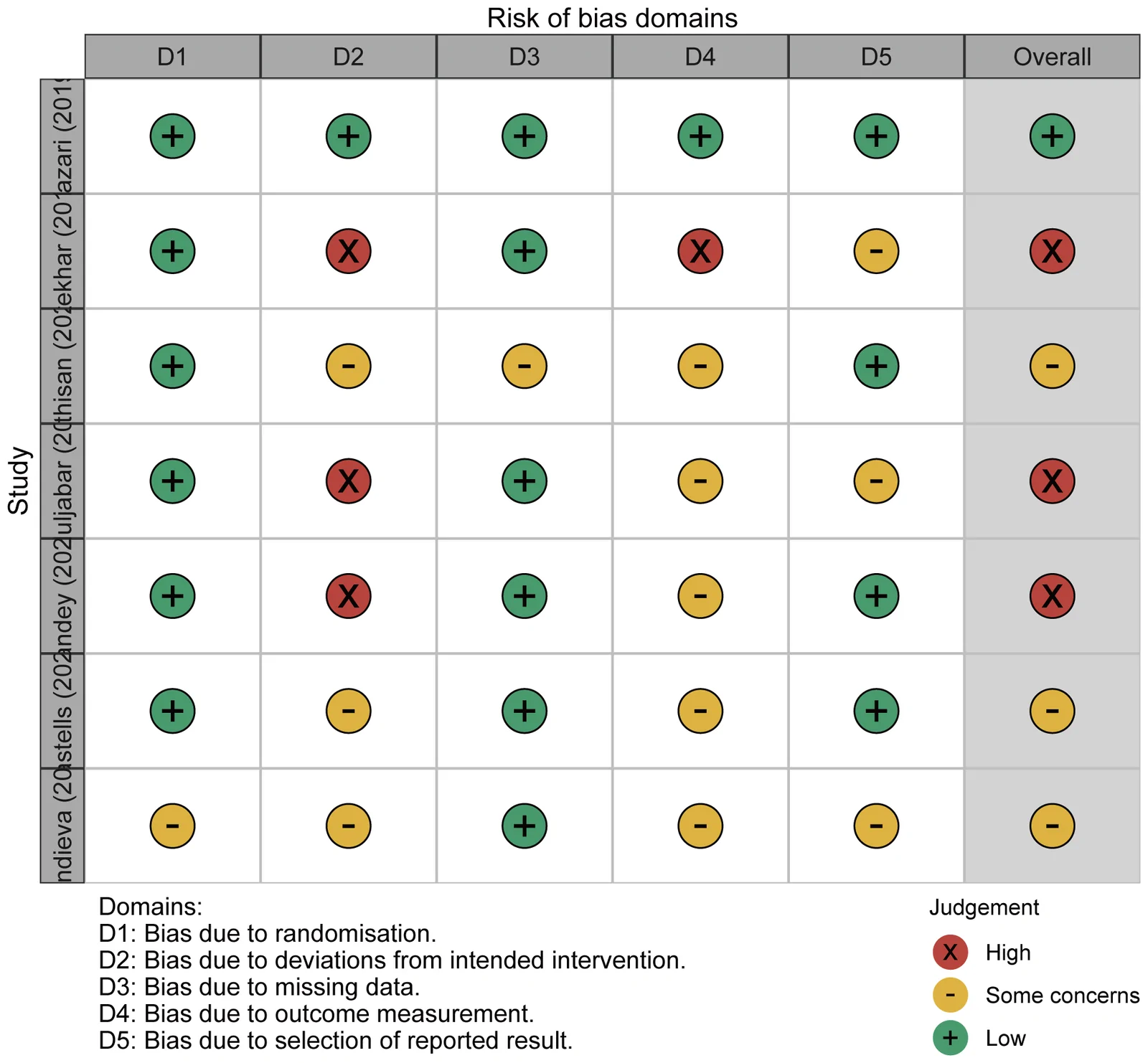
Risk-of-bias (RoB 2) traffic-light plot for the included trials across the five RoB 2 domains and the overall judgment.

**Figure 3:**
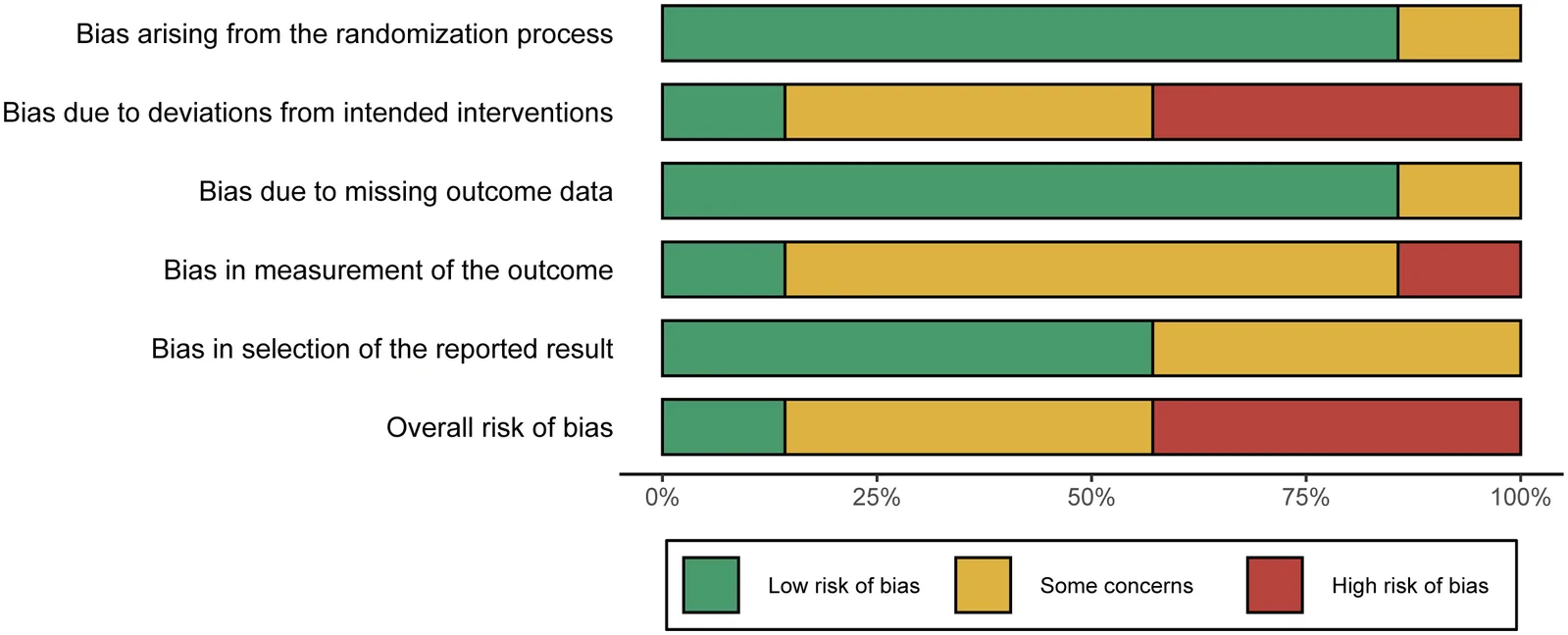
Risk-of-bias (RoB 2) summary bar plot showing the proportion of trials at each level of risk for each domain.

Among the seven trials, only Nazari 2019 was judged to be at low risk of bias across all domains, reflecting its double-blind, sham-controlled design (12). Eftekhar 2018, Abduljabbar 2022, and Pandey 2023 were rated at high overall risk of bias, chiefly owing to the absence of blinding and of a sham control, with concerns concentrated in deviations from intended interventions (D2) and outcome measurement (D4) (13, 14, 16). Pothisan 2025, Castells 2025, and Efendieva 2023 were rated as raising some concerns (15, 29, 30). Domain-level judgments are displayed in **Figure 2**, and the overall distribution in **Figure 3**.

### 3.3 Clinical pregnancy

**Figure 4:**
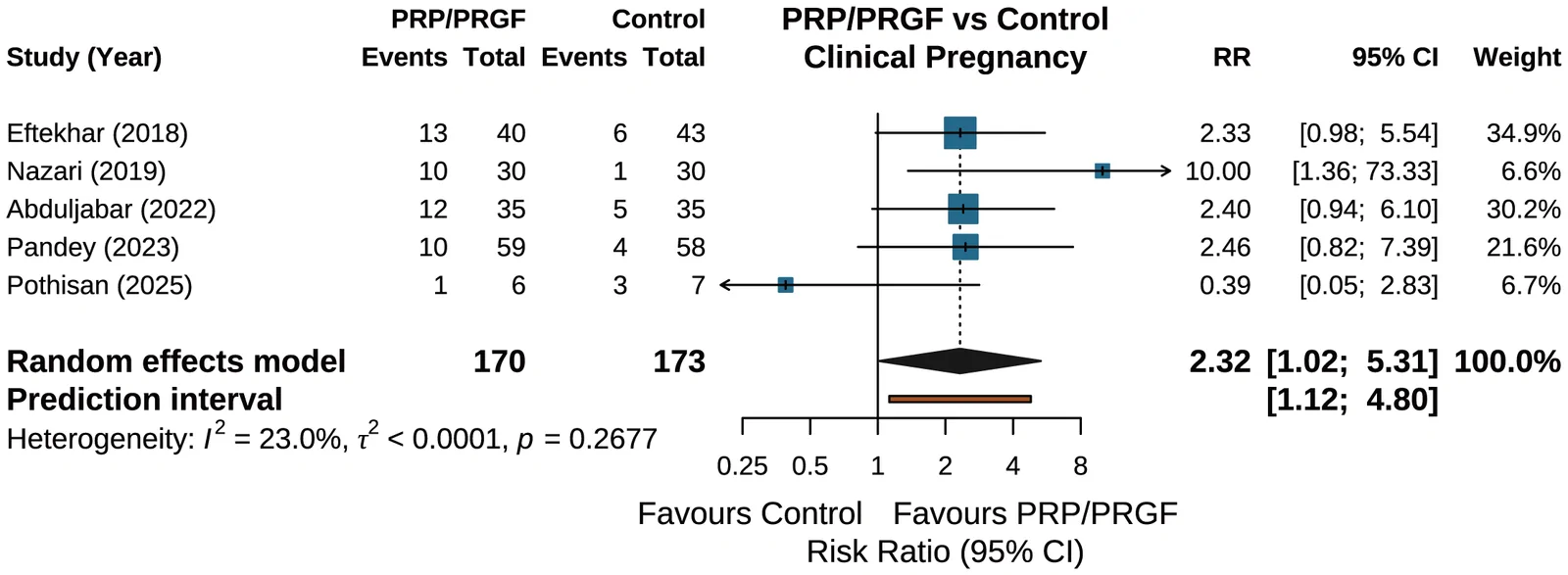
Forest plot comparing clinical pregnancy between PRP/PRGF and control (random-effects model, risk ratio).

Five trials (170 PRP and 173 control participants) reported poolable clinical-pregnancy data (12–16). Under the random-effects model, PRP or PRGF was associated with a higher clinical-pregnancy rate than control, with a pooled RR of 2.32 (95% CI 1.02-5.31; p=0.047) and low heterogeneity (I-squared=23.0%). Among individual trials, the RR was 2.33 for Eftekhar 2018, 2.40 for Abduljabbar 2022, and 2.46 for Pandey 2023; Nazari 2019 showed the largest effect (RR 10.00), whereas Pothisan 2025—small and in the opposite direction (RR 0.39)—carried little weight. The prediction interval was 1.12-4.80 (**Figure 4**).

### 3.4 Subgroup analysis by clinical setting

**Figure 5:**
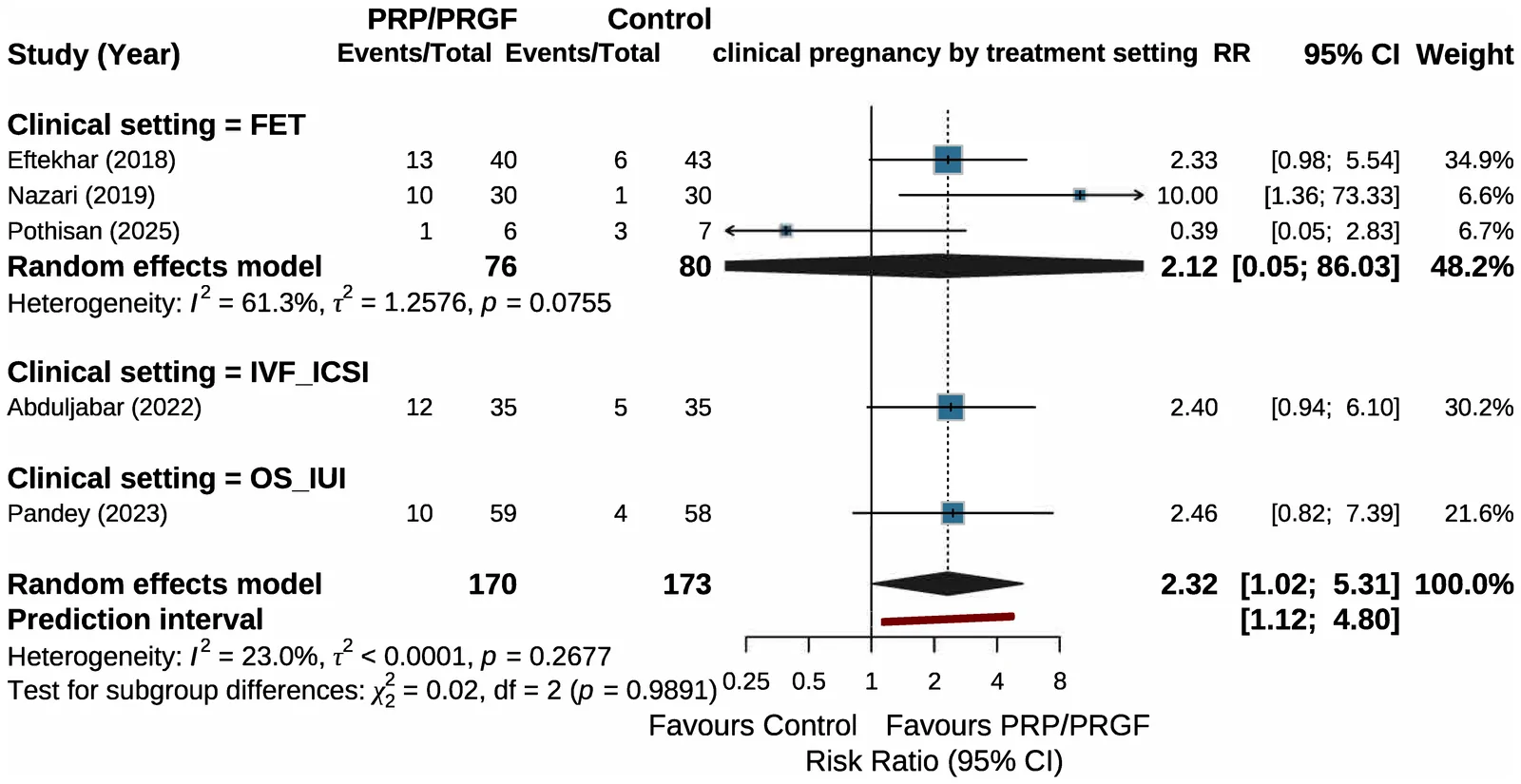
Subgroup forest plot of clinical pregnancy stratified by clinical setting (FET, IVF/ICSI, OS-IUI).

Because the trials spanned FET, IVF/ICSI, and OS-IUI, a subgroup analysis by clinical setting was performed. The test for subgroup differences was not significant (Q = 0.02, df=2, p=0.989). This result does not establish equivalence across settings, because the FET subgroup contained only three trials and the IVF/ICSI and OS-IUI subgroups contained one trial each; it indicates only that no subgroup difference was detected with the available sparse data (**Figure 5**).

### 3.5 Biochemical pregnancy

**Figure 6:**
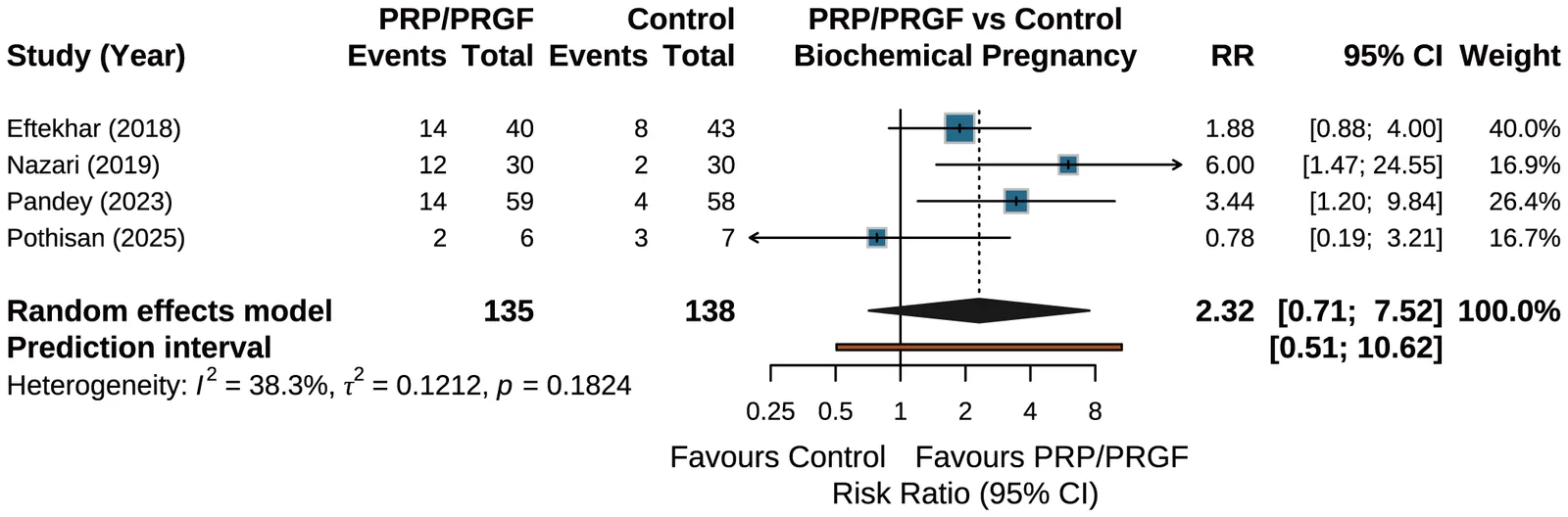
Forest plot comparing biochemical pregnancy between PRP/PRGF and control (random-effects model, risk ratio).

Four trials (135 PRP and 138 control participants) reported biochemical pregnancy (12, 13, 15, 16). The pooled RR was 2.32 (95% CI 0.71-7.52), which after the Hartung-Knapp adjustment did not reach statistical significance (p=0.108), with moderate heterogeneity (I-squared=38.3%). Individual RRs were 1.88 for Eftekhar 2018 and 0.78 for Pothisan 2025, while Nazari 2019 (RR 6.00) and Pandey 2023 (RR 3.44) were individually significant; nonetheless, although the pooled point estimate numerically favored PRP or PRGF, the wide CI crossing the null precluded a firm conclusion (**Figure 6**).

### 3.6 Endometrial thickness change

**Figure 7:**
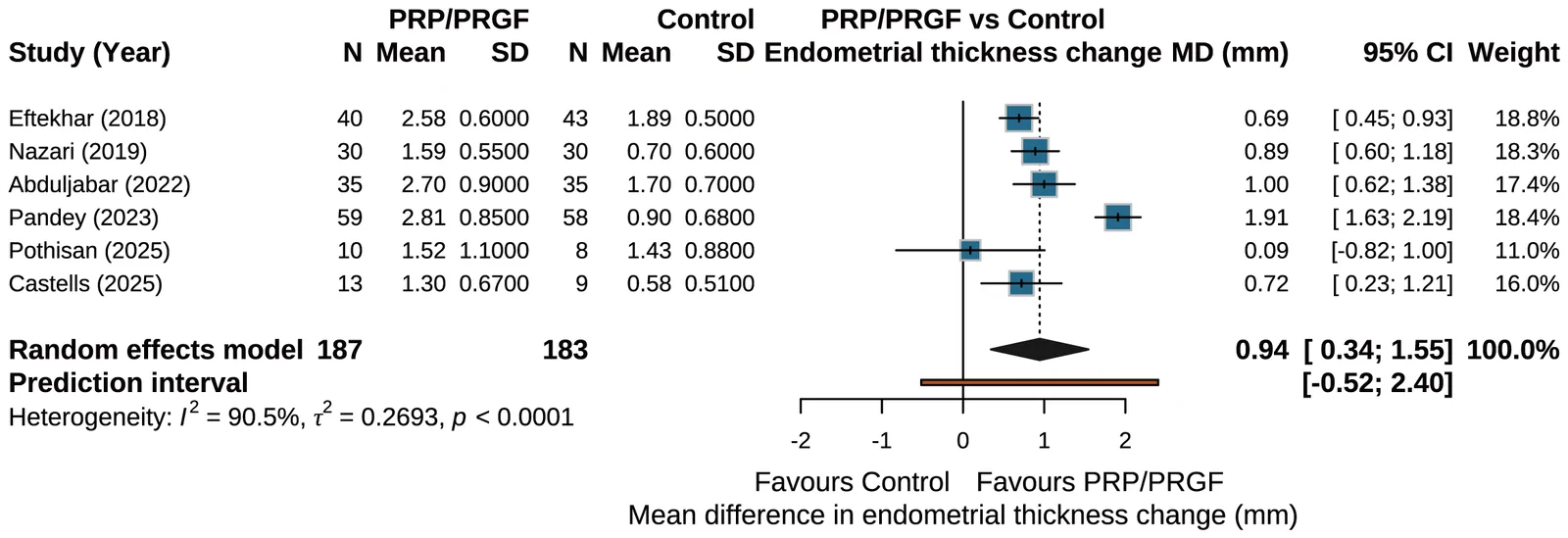
Forest plot comparing endometrial thickness change between PRP/PRGF and control (random-effects model, mean difference in mm).

Six trials (187 PRP and 183 control participants) reported the change in endometrial thickness (12–16, 29). The random-effects model showed a significantly greater increase in the PRP or PRGF group, with a pooled MD of 0.94 mm (95% CI 0.34-1.55; p=0.010). Heterogeneity was very high (I-squared=90.5%), consistent with substantial differences across trials in the timing of measurement and in PRP preparation and infusion protocols. The largest increases were seen in Pandey 2023 (MD 1.91 mm) and Nazari 2019 (MD 0.89 mm), and the smallest between-group difference in Pothisan 2025 (MD 0.09 mm) (**Figure 7**). In the PRP-only sensitivity analysis excluding the PRGF study (Castells 2025), the pooled MD was 0.98 mm (95% CI 0.20-1.76; p=0.026; I-squared=92.1%). The direction and statistical significance were therefore unchanged, although heterogeneity remained very high; this supports robustness to exclusion of PRGF but does not demonstrate biological equivalence between PRP and PRGF.

### 3.7 Cycle cancellation (exploratory)

**Figure 8:**
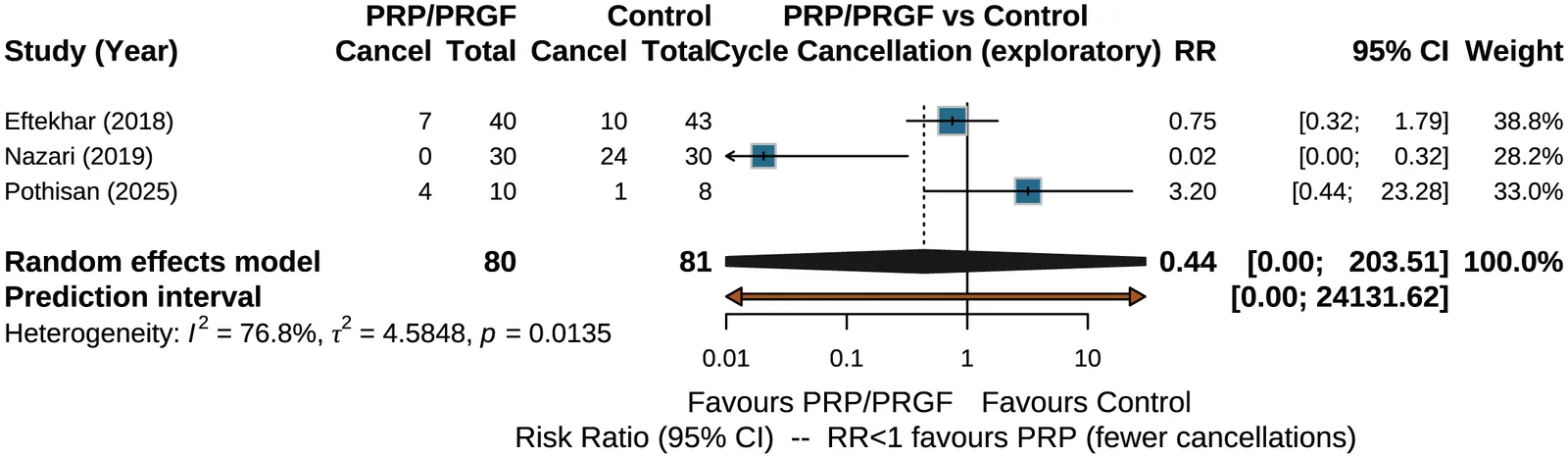
Forest plot comparing cycle cancellation between PRP/PRGF and control (exploratory random-effects model, risk ratio).

Three trials (80 PRP and 81 control participants) reported cycle cancellation (12, 13, 15). The pooled RR was 0.44 (95% CI 0.00-203.51; p=0.622), not significant and with very high heterogeneity (I-squared=76.8%). The three trials pointed in conflicting directions: Eftekhar 2018 gave an RR of 0.75; Nazari 2019, whose sham control cancelled 24 of 30 cycles by design, gave an extreme RR of 0.02; and Pothisan 2025, in which the single-PRP arm was cancelled more often than control, gave an RR of 3.20. This high heterogeneity, driven by fundamental differences in the control condition, means the pooled estimate is only exploratory and cannot support a conclusion that PRP reduces cycle cancellation (**Figure 8**).

### 3.8 Sensitivity analysis

**Figure 9:**
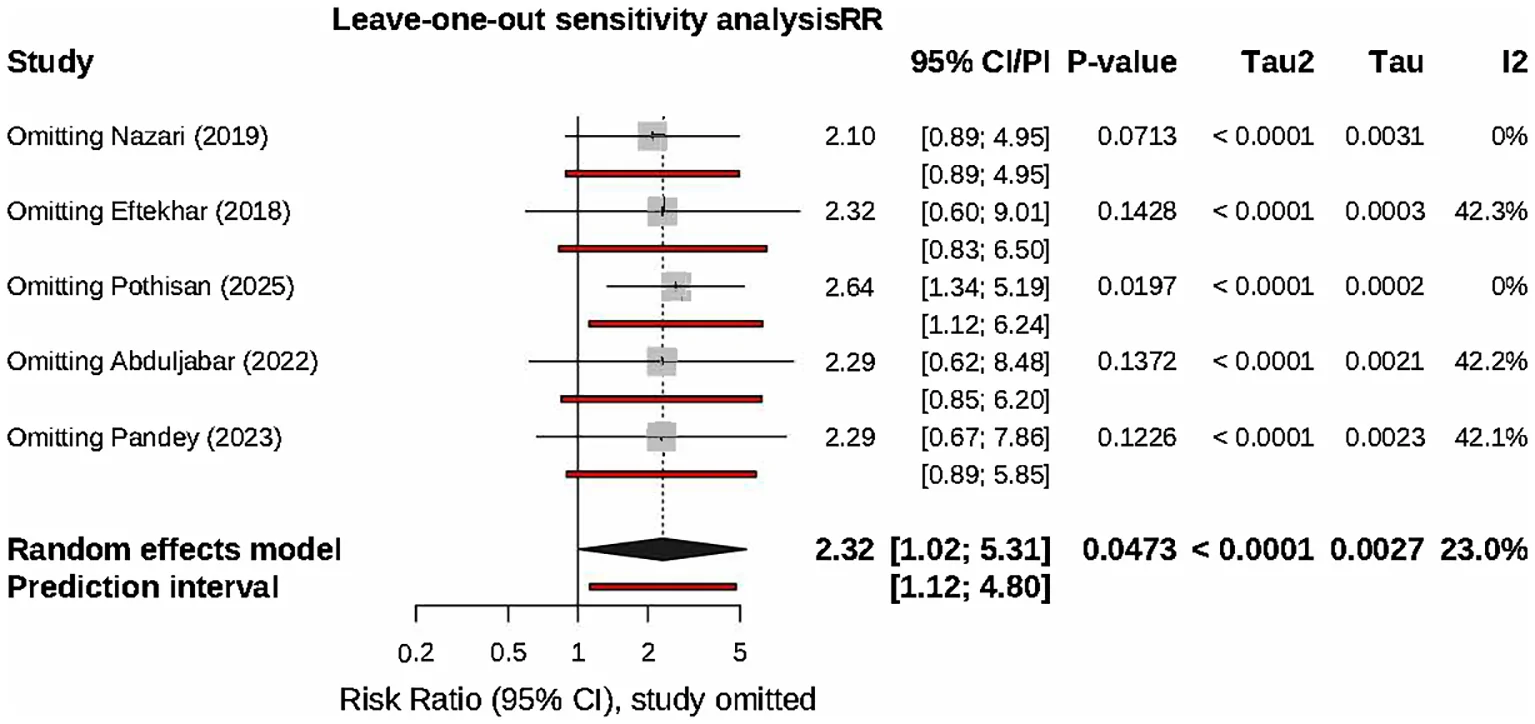
Leave-one-out sensitivity analysis for clinical pregnancy, showing the pooled estimate after omitting each trial in turn.

A leave-one-out sensitivity analysis was performed for clinical pregnancy. The pooled effect was sensitive to individual trials: removing Nazari 2019 (RR 2.10), Eftekhar 2018 (RR 2.32), Abduljabbar 2022 (RR 2.29), or Pandey 2023 (RR 2.29) each caused the 95% CI to cross the null, with the p-value rising to between 0.07 and 0.14, so that the difference was no longer significant; only after removing Pothisan 2025 did the result remain significant (RR 2.64, p=0.020). The positive clinical-pregnancy result therefore rests on several trials jointly rather than on any single one, and is not robust (**Figure 9**).

### 3.9 Small-study effects and influence diagnostics

**Figure 10:**
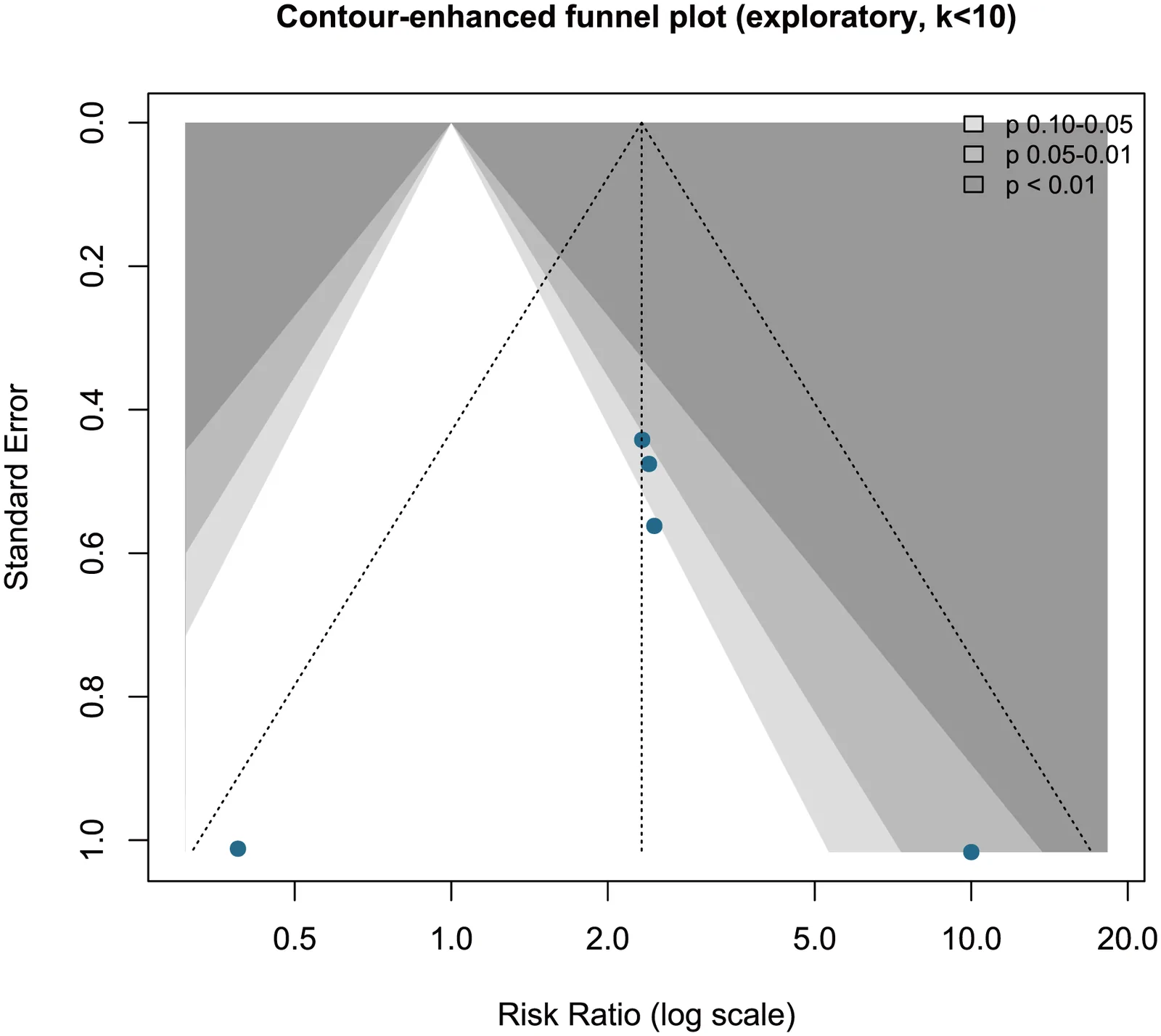
Contour-enhanced funnel plot for clinical pregnancy (exploratory).

**Figure 11:**
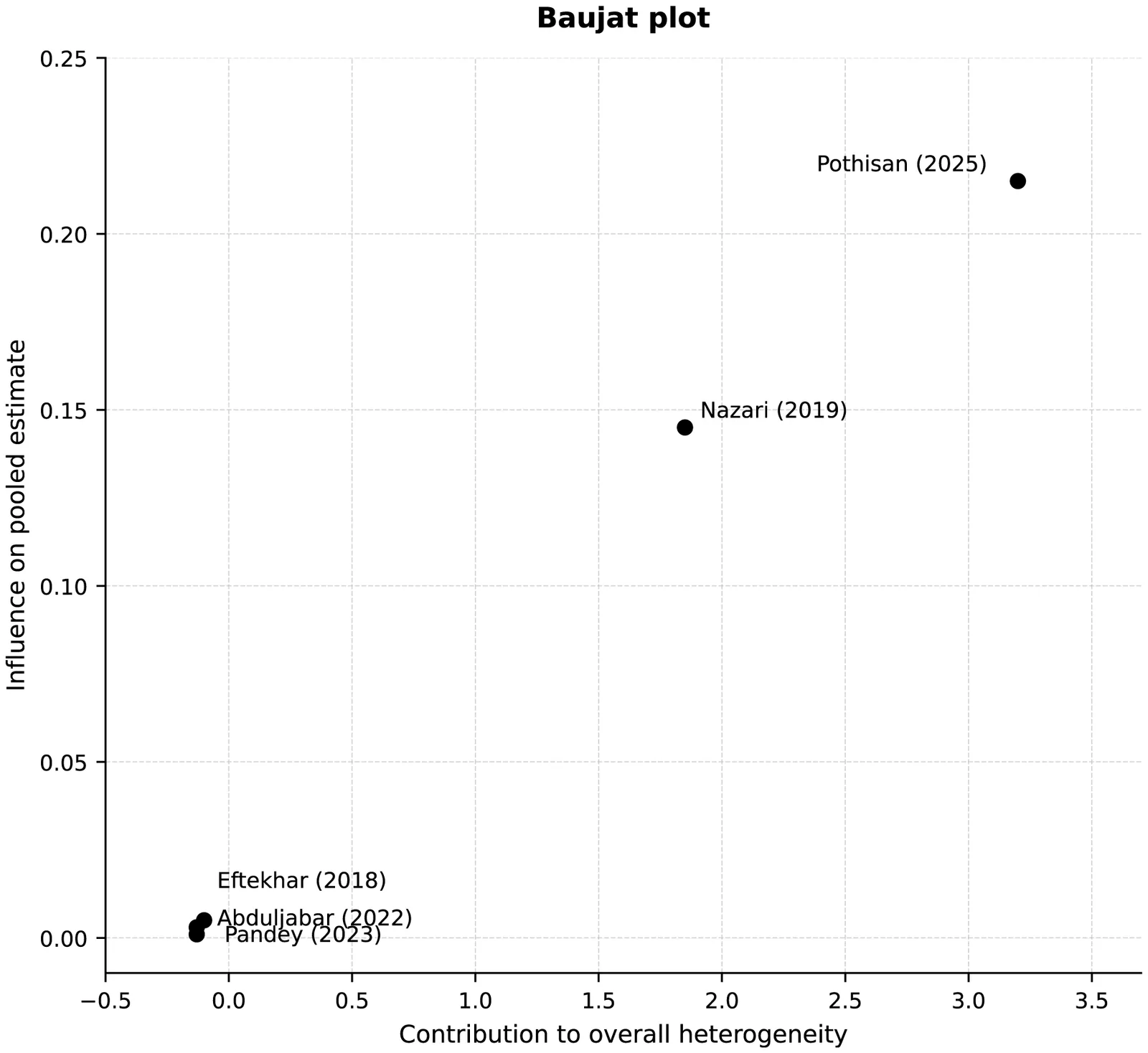
Baujat plot showing each trial’s contribution to heterogeneity (horizontal axis) and influence on the pooled clinical-pregnancy estimate (vertical axis) for the five trials in the primary analysis.

Because fewer than 10 trials contributed to each outcome, tests for small-study effects have limited power and were treated as exploratory. For clinical pregnancy, Egger’s test gave p=0.876 and Begg’s test p=0.327, and the funnel plot showed no marked asymmetry (**Figure 10**). A Baujat plot (7) was used to examine the contribution of the five trials in the primary clinical-pregnancy analysis to overall heterogeneity and to the pooled estimate (**Figure 11**); the results were consistent with the sensitivity analysis, indicating that individual small trials exerted an appreciable influence on the pooled result.

### 3.10 Summary of pooled effects and GRADE certainty of evidence

The pooled effects for all four outcomes are summarized in **Table 2**. In brief, PRP or PRGF significantly increased endometrial thickness and produced a marginally significant improvement in clinical pregnancy, whereas biochemical pregnancy and cycle cancellation did not differ significantly.

**Table 2:** Pooled effect estimates for all outcomes.

Outcome	Trials (n)	Participants	Effect measure	Pooled estimate (95% CI)	p	I-squared
Clinical pregnancy (primary)	5	343	RR (random)	2.32 (1.02 to 5.31)	0.047	23.0%
Biochemical pregnancy	4	273	RR (random)	2.32 (0.71 to 7.52)	0.108	38.3%
Endometrial thickness change	6	370	MD, mm (random)	0.94 (0.34 to 1.55)	0.010	90.5%
Cycle cancellation (exploratory)	3	161	RR (random)	0.44 (0.00 to 203.51)	0.622	76.8%

RR, risk ratio; MD, mean difference; CI, confidence interval. All estimates are from random-effects models (REML) with the Hartung-Knapp adjustment. Cycle cancellation is exploratory (I-squared > 75%): the sham-controlled trial cancels most control cycles by design, so this pooled estimate should be interpreted with caution. Endometrial-thickness change SDs for Nazari 2019, Eftekhar 2018, Pandey 2023, and Abduljabbar 2022 were derived from reported pre-/post-treatment SDs assuming a within-patient correlation r = 0.5.

The certainty of evidence for all assessed outcomes was very low and is summarized in **Table 3**. Clinical pregnancy and endometrial thickness change were downgraded for risk of bias, imprecision, and inconsistency or indirectness; biochemical pregnancy and cycle cancellation were downgraded for wide confidence intervals and heterogeneity. Live birth was not meta-analysed because only two RCTs reported it and the available comparative data were too sparse to support a stable pooled estimate. The evidence base therefore remains insufficient for firm efficacy conclusions.

**Table 3:** GRADE summary of findings for the main outcomes.

Outcome	Trials (n)	Certainty (GRADE)	Reasons for downgrading
Clinical pregnancy	5	Very low	Risk of bias; imprecision; inconsistency of settings
Endometrial thickness change	6	Very low	Risk of bias; indirectness; SD reconstruction
Biochemical pregnancy	4	Very low	Risk of bias; imprecision (wide CI); heterogeneity
Cycle cancellation	3	Very low	Very high heterogeneity; imprecision; sham-control artifact
Live birth (not meta-analysed)	2 RCTs	Very low	Only two RCTs reported live birth; comparative data too sparse for reliable pooling

GRADE certainty: high, moderate, low, or very low. The largest RCT in the field (Elbanna 2023, trial registration NCT03945812, n=390) reported that PRP was non-superior to placebo but remains a conference abstract and could not be pooled; this contributes to the very-low certainty rating.

### 3.11 Studies excluded from quantitative synthesis

Six further studies were identified that did not meet the inclusion criteria, including a recent trial that compared single versus double PRP infusion without a non-PRP control arm (31); the reasons for their exclusion are given in **Table 4**. Of particular note is Elbanna 2023, the largest double-blind RCT in this field to date (n=390), which reported that neither PRP nor granulocyte colony-stimulating factor was superior to placebo for pregnancy outcomes (18). Because it is available only as a conference abstract with no full text at the search date, it could not be included in the quantitative synthesis and is instead considered in the Discussion as important contradictory evidence.

**Table 4:** Studies excluded from the quantitative synthesis, with reasons.

Study (year)	Design	Reason for exclusion
Shin (2024) (42)	Prospective cohort	Not an RCT (within-patient pre/post comparison)
Feng (2025) (31)	Single-blind RCT	No non-PRP control arm (single vs double PRP)
Salman (2023) (43)	Single-arm prospective	No control group
Coksuer (2019) (37)	Retrospective observational	Not an RCT; groups by baseline EMT (selection bias)
Elbanna (2023) (18)	Double-blind RCT (n=390)	Conference abstract only; no full text
Akanksha IVF (2024)	RCT	Conference abstract only; no full text

EMT, endometrial thickness. Elbanna 2023 (trial registration NCT03945812) reported that neither PRP nor G-CSF was superior to placebo for pregnancy outcomes; its abstract-only status at the search date prevented inclusion, but it is discussed as important contradictory evidence.

## 4 Discussion

### 4.1 Principal findings

This systematic review and meta-analysis synthesized the randomized evidence on intrauterine PRP or PRGF for thin endometrium. The clearest finding was that PRP or PRGF increases endometrial thickness, with a significant pooled mean difference of 0.94 mm. For clinical pregnancy, the PRP or PRGF group showed a marginally significant improvement (RR 2.32, p=0.047), whereas no significant difference was observed for biochemical pregnancy or cycle cancellation. The overall pattern is thus one of a clear effect on endometrial thickening alongside only preliminary evidence for improved pregnancy outcomes. This pattern is clinically important because endometrial thickness, although the most accessible marker of the endometrial response, is only a proxy for the receptive capacity that ultimately determines whether an embryo implants. A treatment that reliably thickens the endometrium is therefore encouraging but not sufficient on its own to justify a change in practice; the decisive evidence must come from adequately powered trials reporting pregnancy and live birth. It is also worth noting the hierarchy among the outcomes examined. Endometrial thickness is an intermediate, operator-dependent measure; biochemical pregnancy reflects early implantation but not necessarily a viable gestation; clinical pregnancy, defined by an ultrasound-visible gestational sac, is more meaningful but still short of the outcome that matters most to patients. Live birth, the definitive endpoint, was reported by too few of the included trials to allow reliable pooling. The evidence therefore becomes progressively thinner as one moves from surrogate measures toward clinically decisive ones, and the apparently positive picture at the level of endometrial thickness should not be over-extrapolated to the outcomes that ultimately guide practice. Only two RCTs reported live birth, and their sparse, clinically heterogeneous data were insufficient for a reliable meta-analysis; live birth was therefore summarized narratively rather than treated as a pooled endpoint.

### 4.2 Comparison with previous studies and mechanistic considerations

The biological rationale for intrauterine PRP rests on the concentrated growth factors it delivers to the endometrium. PRGF stimulates the proliferation and migration of endometrial stromal cells and the synthesis of extracellular-matrix components; VEGF is a principal driver of angiogenesis and neovascularization, improving sub-endometrial perfusion; TGF-beta and EGF modulate epithelial regeneration and immune balance; and IGF-1 supports cell survival and proliferation (4–6, 32). Most of these factors have cognate receptors on endometrial epithelial and stromal cells, so their local delivery can plausibly promote endometrial remodeling and enhance receptivity (6, 32). Transcriptomic studies of human endometrial stromal cells exposed to autologous PRP have reported changes in the expression of genes linked to epithelial regeneration, angiogenesis, and cell signaling, offering a molecular correlate for the clinical observations (32). PRP has also been reported to upregulate adhesion molecules and receptivity-related genes and to recruit stem or progenitor cells to the endometrium, mechanisms that extend beyond a simple increase in thickness (32, 33). Our pooled finding of a significant increase in endometrial thickness is consistent with this proangiogenic, proliferative mechanism and with numerous single-center observations (5, 34). Whether that increase translates into better pregnancy outcomes, however, remains insufficiently supported by the randomized evidence. This dissociation between endometrial thickening and pregnancy is instructive: it suggests that receptivity depends not only on thickness but also on the functional and vascular state of the endometrium and on molecular determinants of the implantation window that a growth-factor concentrate may not fully restore (1, 2, 30). Comparable dissociations—improved thickness or perfusion without a corresponding rise in live birth—have been reported both for PRP and for other endometrial interventions (31, 33, 35, 36).

### 4.3 Consideration of contradictory evidence

These positive results have to be read against the contradictory evidence in the field. The largest double-blind RCT to date (Elbanna 2023, n=390) found that PRP was not superior to saline placebo for clinical pregnancy; although PRP improved endometrial perfusion, this did not translate into a higher pregnancy rate (18). Because this trial is so far available only as a conference abstract and lacks complete data, it could not be incorporated into the present synthesis. This large negative trial is a strong reason for caution when interpreting the positive results from small studies; if it were formally published and pooled, it could attenuate or even reverse the current estimate. Trials of PRP in related indications such as recurrent implantation failure have likewise yielded mixed results (37–40), and previous broader meta-analyses have reached only tentative conclusions (19, 20). The discrepancy between the encouraging signal in small trials and the null result of the largest trial is a familiar pattern in evidence synthesis, in which early small studies tend to report larger effects that regress toward the null as larger and more rigorous trials accumulate. This possibility should temper enthusiasm and argues against adopting PRP as standard care on the basis of the current evidence. It also highlights the importance of publishing the full results of completed but unreported trials, whose absence from the literature may bias the available evidence toward a favorable impression.

### 4.4 Robustness and heterogeneity

Our findings can be placed alongside earlier syntheses. Broader meta-analyses of intrauterine PRP in assisted reproduction—which pooled mixed populations of recurrent implantation failure and thin endometrium—reported favorable effects on pregnancy, but with the same caveats of small, heterogeneous, and largely unblinded trials (14, 15, 41). By restricting eligibility to randomized trials in thin endometrium and analyzing each outcome separately, the present review isolates a more specific question and reaches a more tempered conclusion: a clear effect on thickness, a fragile signal for clinical pregnancy, and no demonstrable effect on biochemical pregnancy or cycle cancellation. The leave-one-out analysis makes the fragility explicit: removing any of several individual trials abolished statistical significance for clinical pregnancy, so the positive result rests on the joint contribution of several trials rather than on any single high-quality study. The pooled estimate for endometrial thickness change also carried very high heterogeneity (I-squared=90.5%), as did that for cycle cancellation (I-squared=76.8%). Potential sources of heterogeneity include differing definitions of thin endometrium (most below 7 mm, but Castells 2025 required below 5 mm), differences in clinical setting (FET, IVF/ICSI, and OS-IUI combined), variation in PRP preparation and infusion protocols (volume, number of infusions, and activation method), and fundamental differences in the control condition. Cycle cancellation is the clearest example: the sham-controlled Nazari 2019 cancelled most control cycles by design, whereas in Pothisan 2025 the single-PRP arm was cancelled more often than control, the two pointing in opposite directions and rendering the pooled estimate uninterpretable. Non-randomized reports and single-arm series (42, 43) cannot resolve this uncertainty, underscoring the value of adhering to standardized synthesis methods (22). Beyond the control condition, the PRP preparations themselves varied in ways that are rarely reported in full. Trials differed in the centrifugation protocol, the resulting platelet concentration, whether leukocytes were retained or depleted, the activating agent, the infused volume, and the number and timing of infusions relative to the transfer cycle. Each of these parameters can alter the dose and release kinetics of the delivered growth factors, so that nominally identical PRP interventions may not be biologically equivalent across studies. This lack of standardization is a plausible contributor to the very high heterogeneity observed for endometrial thickness and limits the extent to which a single pooled estimate can be interpreted as a property of PRP in general rather than of the particular preparations tested. The PRP-only sensitivity analysis produced a similar endometrial-thickness estimate after exclusion of the PRGF study, supporting the direction of the pooled result under the pragmatic class-effect assumption while not implying that the products are identical. Likewise, the setting subgroup test was non-significant (p=0.989), but the single-trial IVF/ICSI and OS-IUI strata were too sparse for this to be interpreted as evidence of equivalence across populations.

### 4.5 Implications for clinical practice

On current evidence, PRP or PRGF may be regarded as an exploratory adjunct for increasing endometrial thickness in women with thin endometrium, but it is not yet sufficient to support a conclusion that it reliably improves pregnancy outcomes. Because the certainty of evidence is generally low (very low for most outcomes), patients should be fully informed of the limitations of the current evidence, and the balance of benefit and cost should be weighed carefully. The observation in Pothisan 2025 that a second PRP infusion was accompanied by a higher cycle-cancellation rate further suggests that increasing the number of infusions does not necessarily confer additional benefit, and that the optimal infusion protocol remains to be defined. From a shared-decision-making perspective, PRP may be most reasonably considered for women with truly refractory thin endometrium who have exhausted conventional options and who understand that any benefit for live birth is unproven. Counseling should make clear that the most reliable effect is on endometrial thickness, that the effect on clinical pregnancy is marginal and statistically fragile, and that the largest trial to date found no advantage over placebo. Because PRP is autologous and prepared from the patient’s own blood, its safety profile appears favorable and the direct material cost is modest; however, the procedure adds venepuncture, processing time, and an additional intrauterine instillation to the treatment cycle, and these burdens should be weighed against an uncertain probability of benefit. In settings where resources are constrained, the opportunity cost of an unproven adjunct may be an important consideration.

### 4.6 Directions for future research

Several priorities emerge for future work. First, adequately powered, double-blind, sham-controlled randomized trials are needed, since blinding and a sham procedure were the features that most clearly distinguished the single low-risk trial in this review from the remainder (8, 17). Second, the definition of thin endometrium and the timing of its measurement should be standardized, because differing thresholds and measurement points were an important source of heterogeneity. Third, PRP preparation and administration should be harmonized and reported in detail—platelet concentration, leukocyte content, activation method, infused volume, number and timing of infusions, and route (transcervical instillation versus sub-endometrial or hysteroscopic injection)—so that trials can be meaningfully compared and an optimal protocol identified (6, 7). Fourth, trials should report a common core outcome set that prioritizes live birth alongside clinical pregnancy, together with the systematic ascertainment of adverse events, rather than surrogate endpoints such as endometrial thickness alone. Finally, individual-participant-data meta-analysis and prospective registration of ongoing trials would help resolve the fragility of the current estimates and clarify which subgroups, if any, derive genuine benefit. An informative future trial would enrol women with a clearly defined refractory thin endometrium, randomize them to intrauterine PRP or a sham instillation under double-blind conditions, fix and report a single standardized PRP preparation and dosing schedule, and follow participants to live birth with prospective ascertainment of adverse events. Stratification by clinical setting and by the underlying cause of thin endometrium would help identify any subgroup in which the intervention is truly effective. Until such trials are available, the role of PRP in thin endometrium will remain promising but unproven.

### 4.7 Strengths and limitations

This review has several strengths. It addressed a focused, clinically relevant question by restricting eligibility to randomized trials in women with thin endometrium, rather than pooling heterogeneous indications; it analyzed clinically distinct outcomes separately using appropriate effect measures; it applied the current Cochrane RoB 2 tool and the GRADE framework; and it examined the robustness of the primary result through leave-one-out and influence diagnostics. The reference list was screened for relevance to reproductive medicine and evidence-synthesis methodology, and the pooled estimates are reported transparently with their prediction intervals and heterogeneity. Nonetheless, important limitations remain. First, the number of included trials was limited and most had modest samples, so the pooled estimates have limited power; we therefore used random-effects models with the Hartung-Knapp adjustment to avoid overconfidence. Second, most included trials were open-label or non-blinded, giving a generally high risk of bias. Third, the SD of endometrial thickness change in several trials was imputed assuming a correlation of 0.5 rather than reported directly; although the sensitivity analysis showed this did not change the direction of the conclusion, it may introduce some uncertainty. Fourth, the largest negative RCT could not be included because it is available only as an abstract, which may render the current pooled estimate overly optimistic. Fifth, fewer than 10 trials contributed to every outcome, so tests for small-study effects had limited power. Together, these limitations account for the low certainty of the evidence. Sixth, several trials reported outcomes as medians or did not provide the variances needed for pooling, requiring assumptions during data extraction that introduce additional uncertainty. Seventh, the included trials originated from a small number of centers in a limited set of countries, which may constrain the generalizability of the findings to other populations and laboratory settings. Eighth, live birth—the outcome of greatest importance to patients—was reported by too few trials to support a reliable pooled estimate, so our conclusions rest on intermediate endpoints. These considerations reinforce that the present results should be read as hypothesis-generating rather than definitive.

### 4.8 Conclusion

The available randomized evidence indicates that intrauterine infusion of PRP or PRGF effectively increases endometrial thickness in women with thin endometrium and is associated with a marginally significant improvement in clinical pregnancy, but without demonstrable benefit for biochemical pregnancy or cycle cancellation. Constrained by the small number of trials, high risk of bias, and low certainty of evidence, these conclusions remain preliminary. Larger, rigorously designed, multicenter RCTs with standardized definitions of thin endometrium and standardized PRP infusion protocols are needed to establish the true value of PRP or PRGF for improving pregnancy outcomes.

## Data Availability

The original contributions presented in the study are included in the article/supplementary material. Further inquiries can be directed to the corresponding author.
